# Hot spring distribution and survival mechanisms of thermophilic comammox *Nitrospira*

**DOI:** 10.1038/s41396-023-01409-w

**Published:** 2023-04-17

**Authors:** Yan Zhang, Tao Liu, Meng-Meng Li, Zheng-Shuang Hua, Paul Evans, Yanni Qu, Sha Tan, Min Zheng, Hui Lu, Jian-Yu Jiao, Sebastian Lücker, Holger Daims, Wen-Jun Li, Jianhua Guo

**Affiliations:** 1 School of Environmental and Chemical Engineering, Foshan University, Foshan, China; 2 Australian Centre for Water and Environmental Biotechnology, Faculty of Engineering, Architecture and Information Technology, The University of Queensland, St Lucia, QLD Australia; 3 State Key Laboratory of Biocontrol, Guangdong Provincial Key Laboratory of Plant Resources and Southern Marine Science and Engineering Guangdong Laboratory (Zhuhai), School of Life Sciences, Sun Yat-sen University, Guangzhou, China; 4 Department of Environmental Science and Engineering, University of Science and Technology of China, Hefei, China; 5 The Australian Centre for Ecogenomics, School of Chemistry and Molecular Biosciences, University of Queensland, St Lucia, QLD Australia; 6 School of Environmental Science and Engineering, Sun Yat-sen University, Guangzhou, 510275 China; 7 Department of Microbiology, RIBES, Radboud University, Heyendaalseweg 135, 6525 AJ Nijmegen, the Netherlands; 8 Division of Microbial Ecology, Centre for Microbiology and Environmental Systems Science, University of Vienna, Djerassiplatz 1, 1030 Vienna, Austria; 9 The Comammox Research Platform, University of Vienna, Djerassiplatz 1, 1030 Vienna, Austria

**Keywords:** Biogeography, Microbial ecology, Water microbiology

## Abstract

The recent discovery of *Nitrospira* species capable of complete ammonia oxidation (comammox) in non-marine natural and engineered ecosystems under mesothermal conditions has changed our understanding of microbial nitrification. However, little is known about the occurrence of comammox bacteria or their ability to survive in moderately thermal and/or hyperthermal habitats. Here, we report the wide distribution of comammox *Nitrospira* in five terrestrial hot springs at temperatures ranging from 36 to 80°C and provide metagenome-assembled genomes of 11 new comammox strains. Interestingly, the identification of dissimilatory nitrate reduction to ammonium (DNRA) in thermophilic comammox *Nitrospira* lineages suggests that they have versatile ecological functions as both sinks and sources of ammonia, in contrast to the described mesophilic comammox lineages, which lack the DNRA pathway. Furthermore, the in situ expression of key genes associated with nitrogen metabolism, thermal adaptation, and oxidative stress confirmed their ability to survive in the studied hot springs and their contribution to nitrification in these environments. Additionally, the smaller genome size and higher GC content, less polar and more charged amino acids in usage profiles, and the expression of a large number of heat shock proteins compared to mesophilic comammox strains presumably confer tolerance to thermal stress. These novel insights into the occurrence, metabolic activity, and adaptation of comammox *Nitrospira* in thermal habitats further expand our understanding of the global distribution of comammox *Nitrospira* and have significant implications for how these unique microorganisms have evolved thermal tolerance strategies.

## Introduction

Over the last century, chemolithoautotrophic nitrification was believed to be a two-step process carried out by a consortium of microorganisms. However, this paradigm has been radically changed by epoch-making discoveries of single organisms able to oxidize ammonia to nitrate [1, 2]. These chemolithoautotrophic complete ammonia-oxidizing (comammox) bacteria belong to sublineage II of the deep-branching genus *Nitrospira* previously considered to only perform nitrite oxidation [3]. While attempts have been made to fully characterize comammox *Nitrospira*, currently only one isolated strain and a limited number of enrichment cultures have been obtained [4–7]. From two of these comammox *Nitrospira* cultures, kinetic characterization suggests that they have a high affinity for ammonia and prefer to inhabit oligotrophic environments [4, 5]. Thus, ecosystems with limited nutrient flow favor the presence of comammox *Nitrospira*, which may even dominate over canonical ammonia oxidizers [e.g., ammonia-oxidizing bacteria (AOB)]. However, field studies have suggested that factors other than ammonia availability will also play a role in the niche differentiation of ammonia oxidizers [8, 9].

The ecological significance of comammox *Nitrospira* has only been partially deciphered, although they have been found to be widely distributed in non-marine natural and engineered ecosystems. Environments, where they have been detected, include soils, natural freshwater settings, wastewater treatment installations, and drinking water production and distribution systems [10–18]. Except for the first and so far only isolated comammox strain *Nitrospira inopinata*, which was originally sampled from a hot water pipe (56°C) and cultured under moderately thermal conditions at 46 to 50 °C [1, 4], all other studies were associated with mesothermal conditions. This raises the question about the role comammox *Nitrospira* play in nitrogen cycling in thermal and hyperthermal habitats.

Geothermal habitats like submarine hydrothermal vents and terrestrial hot springs support a variety of chemolithoautotrophic microorganisms, possibly since the origin of cellular life [19–21]. Investigations of hot spring microbial communities can be traced back to the 19th century [22], while the understanding of their diversity and metabolic capacity has been significantly improved in the last two decades due to the advances in sequencing technologies. One compound in hot springs that is of particular interest is ammonium, which is the major nitrogenous compound observed in geothermal habitats [23]. Consequently, thermophilic aerobic ammonia-oxidizing archaea (AOA) have been widely found in thermal ecosystems [24–27]. Additionally, nitrite-oxidizing bacteria (NOB) affiliated with the genus *Nitrospira* or the phylum *Chloroflexi* have been identified in geothermal hot springs [28–30]. The potential for members of the genus *Nitrospira*, including the comammox species *N. inopinata*, to tolerate thermally induced stress indicates that also comammox *Nitrospira* may proliferate in high-temperature ecosystems, but no such report has been made to date for natural geothermal springs. This leads to questions of how widely distributed thermophilic comammox *Nitrospira* are, what strategies these species employ to protect themselves from heat stress, and if there are any genomic differences between comammox *Nitrospira* in thermal and non-thermal habitats.

Here, we report the occurrence and activity of comammox *Nitrospira* in terrestrial hot spring ecosystems, explore their potential survival mechanisms under (hyper)thermal conditions, and compare genomic features of comammox *Nitrospira* obtained from thermal and non-thermal habitats. These novel insights into the occurrence, activity, and adaptation of comammox *Nitrospira* in thermal habitats further expand our understanding of the global distribution of these unique microorganisms and provide important insights into microbial heat stress defense strategies.

## Materials and Methods

### Sample collection, DNA extraction, and metagenomic sequencing

A total of 10 biomass samples were collected for DNA extraction from the surface sediments (~top 5 cm) of five hot springs JinZe (JZ), ShuiReBaoZha (SRBZ), and ShiZiTouDuiMian (SZTDM) located at Tengchong county in Yunnan province, and DaGeJia (DGJ) and QuZhuoMu (QZM) located near Rikaze City in Tibet, China. All hot springs samples are at altitudes of ≥ 4000 m above sea level. At the time of sampling, these hot springs spanned a wide range of physicochemical parameters with temperatures and pH ranging from 36.7 to 80.0 °C and 3.0 to 8.2, respectively. Sample locations, sampling dates, and physicochemical parameters are summarized in Supplementary Table S1. The detailed methods for sample collection, chemical analyses, DNA extraction, and metagenomic sequencing are described in a previous study [31].

### Metagenomic assembly and genome binning

Raw metagenomic reads were generated using a Hiseq 4000 instrument (Illumina, USA) and high-quality reads were obtained according to the procedures described previously [32]. Filtered reads were assembled subsequently using SPAdes v3.10.1 [33] with the following options: −k 21,33,55,77,99,111 –meta. Contigs with length >2500 bp were included for genome binning based on tetranucleotide frequency and sequencing depth using MetaBAT v2.12.1 [34]. To calculate sequencing depth, filtered reads were mapped to assembled contigs using BBMap v38.85 using a minimal identity setting of 0.97. The “jgi_summarize_bam_contig_depths” command in MetaBAT was performed to generate the sequencing depth table. Genomic bins were visualized using emergent self-organizing maps (ESOM) [35]. Completeness, contamination, and strain heterogeneity of each metagenome-assembled genome (MAG) were estimated using CheckM v1.0.5 [36]. To reduce contamination and heterogeneity, all MAGs were further manually examined to remove contigs with abnormal coverage or discordant positions within the ESOM. Finally, clean reads of each MAG were recruited using BBMap and reassembled using SPAdes v3.9.0 with parameters: −k 21,33,55,77,99,127 –careful.

### Taxonomy and abundance of MAGs

Taxonomic assignment of the obtained MAGs was performed using GTDB-tk v1.7.0 [37]. The pairwise average amino acid identity (AAI) between MAGs and selected reference genomes was calculated as previously described [38]. The relative abundance of the retrieved MAGs was calculated using coverage information by dividing the sum of coverages for all contigs belonging to the respective MAG by the sum of coverages for all contigs, multiplied by 100. To compare the relative abundance of comammox *Nitrospira* with other populations such as AOB, AOA, and anammox bacteria, MAGs with the possession of any of the following genes were taken into consideration: ammonia monooxygenase (*amoCAB*), hydroxylamine dehydrogenase (*hao*), NO-forming nitrite reductase (*nirS* or *nirK*), hydrazine synthase (*hzs*), hydrazine dehydrogenase (*hdh*), and nitrite oxidoreductase (*nxrAB*).

### Genome annotation and metabolic reconstruction

Protein coding sequences (CDS) were predicted using Prodigal v2.6.3 [39]. Functional annotation and metabolic pathway reconstruction were conducted by searching the predicted CDS against databases including NCBI non-redundant (nr), evolutionary genealogy of genes: Non-supervised Orthologous Groups (eggNOG), Kyoto Encyclopedia of Genes and Genomes (KEGG), and Carbohydrate-Active enZYmes (CAZy) using DIAMOND v0.7.9 with an E-value cut-off of < 1e^−5^ [40]. Putative hydrogenases belonging to PF00374 and PF02906 were identified by searching against the Pfam database using “hmmscan” command in HMMer v3.3.2 [41]. The amino acid sequences of putative hydrogenases were uploaded to the web tool HydDB (https://services.birc.au.dk/hyddb/) to determine the subtype classification and to predict possible functions. Amino acid usage and the IVYWREL index for each MAG were computed using custom-made Perl scripts (10.6084/m9.figshare.22220173). Transmembrane helices in proteins were predicted using the online tool TMHMM-2.0 (https://services.healthtech.dtu.dk/service.php?TMHMM-2.0). The isoelectric point (pI) of each protein was calculated using the online tool IPC v2.0 (http://isoelectric.org/index.html).

### Phylogenomic and phylogenetic analysis

To understand the phylogenomic relationships of the hot spring comammox *Nitrospira*, a set of 120 conserved marker genes was retrieved from 131 representative *Nitrospira* genomes from the NCBI Refseq database (Supplementary Dataset 1). The extracted sequences were aligned using MUSCLE v3.8.31 [42], and poorly aligned regions were removed using TrimAl v1.4.rev22 [43]. The phylogenomic tree was generated using IQ-TREE v1.6.10 [44] with ultrafast bootstrapping (-bb 1000), as well as a Shimodaira–Hasegawa–like approximate likelihood-ratio test (SH-aLRT, -alrt 1000). The best model of the tree was LG + F + R6, as determined by ModelFinder [45], and was well supported by the Bayesian Information Criterion.

For functional proteins (AmoCAB, NxrAB, and the different hydrogenase types), amino acid sequences were aligned and filtered. Phylogenies were constructed with the same procedures as described above. The best models for the AmoCAB, NxrAB, and hydrogenase-based phylogenies (based on the large subunits) were LG + F + R4, LG + F + G4, and LG + R9, respectively. These models were all well supported by the Bayesian Information Criterion. The generated maximum-likelihood trees were visualized using iTOL v6 (https://itol.embl.de).

### Metatranscriptomic analysis

The in situ metabolic activity of comammox *Nitrospira* in hot springs was explored using metatranscriptomic sequencing to determine which of their genes were actively expressed. Due to difficulties with preserving RNA of sufficient quality from these extreme thermal environments, RNA was successfully extracted only from only one sample from QiaoQuan (QQ) in July 2022 and two samples from JinZe (JZ-2 and JZ-4) in Jan 2021 (Supplementary Table S1). Total RNA was extracted using the RNeasy PowerSoil Total RNA kit (QIAGEN) and 20–30 Gbps of raw sequence data was generated using a HiSeq 4000 instrument at Guangdong Magigene Biotechnology Co., Ltd. (Guangzhou, China). Raw reads were quality controlled using Sickle v1.33 (https://github.com/najoshi/sickle) with the parameters -t sanger –quiet -l 50. Seven of the 11 hot spring comammox MAGs were manually selected as reference genomes according to AAI ≥ 95% at the whole genome level (Supplementary Table S2). Clean reads were mapped onto reference genomes using BBMap v35.85 with the same parameters as above. The interconversions between the.bam and.bed file formats were carried out using SAMTools v1.3.1 [46] and BEDTools v2.30.0 [47]. Gene expression levels in fragments per kilobase of transcript per million fragments mapped (FPKM) were calculated using Cufflinks v2.2.1 [48] for each sample.

## Results and discussion

### Assembly of eleven comammox *Nitrospira* genomes from hot springs

Ten metagenomes were generated from sediment samples collected from five hot springs located in Tengchong (Yunnan Province) and Angren (Tibet Autonomous Region), China (Supplementary Table S1). Temperatures at the sampling locations ranged from 36.7 to 80.0 °C, while the pH varied from 3.0 to 8.2. Ammonium concentrations in examined sediments were mostly below 10 mg N/kg (0.7 mmol/kg), except for the sample taken from ShiZiTouDuiMian hot spring (16.3 mg N/kg, 1.2 mmol/kg).

A total of 11 comammox *Nitrospira* MAGs were successfully reconstructed (Table 1 and Supplementary Table S2). Specifically, four comammox *Nitrospira* MAGs were assembled from two different locations in JinZe hot spring (named JZ-3 and JZ-4), two from DaGeJia (DGJ01_4 and DGJ02_3), two from ShuiReBaoZha (SRBZ-2), two from QuZhuoMu (QZM_B4_2 and QZM_B4_3), and one from ShiZiTouDuiMian (SZTDM-2). These 11 high-quality comammox *Nitrospira* MAGs range in size from 2.46 to 2.95 Mbp with completeness estimates > 88% and contamination values < 3% (Table 1). For the DGJ01_4_Bin137 and DGJ02_3_Bin65 comammox *Nitrospira*, these MAGs were highly similar to each other (99.8% AAI), but stemmed from hot springs with disparate environments in terms of pH and temperature parameters (Supplementary Table S1). The gene expression patterns for DGJ02_3_Bin65 in three samples with pH of 6.8 to 8.6 and temperature of 27 to 70 °C (see below) indicated that these two strains were active in neutral to slightly alkaline environments at a broad temperature range. Whether they can also survive in acidic conditions remains unclear and could only be verified by cultivation-based experiments.

**Table 1:** Characteristics of 11 comammox *Nitrospira* reconstructed from the hot-spring metagenomes.

Bins	Genome size (Mbp)	Comp. (%)^a^	Cont. (%)^a^	No. of contigs	GC (%)	N50 (bp)	No. of rRNAs	No. of tRNAs	OGT (°C)
DGJ01_4_Bin137	2.83	89.7	1.8	95	57.9	48,129	0	39	39.1
DGJ02_3_Bin65	2.86	94.0	1.8	50	57.7	85,185	0	43	38.8
JZ-3_Bin7	2.95	91.4	3.0	9	58.9	507,467	3	35	37.4
JZ-4_Bin17	2.92	90.4	2.3	109	59.3	46,441	1	39	36.5
JZ-4_Bin198	2.72	88.1	0.0	205	59.0	21,200	3	45	37.4
JZ-4_Bin299	2.82	89.0	2.7	119	59.1	38,288	1	40	37.5
QZM_B4_2_Bin429	2.44	93.1	0.0	149	55.5	27,010	0	38	41.9
QZM_B4_3_Bin89	2.52	93.6	1.8	205	58.5	18,544	2	36	39.3
SRBZ-2_Bin184	2.64	92.3	1.8	115	59.0	56,304	0	34	37.2
SRBZ-2_Bin320	2.85	88.9	0.9	26	57.4	279,105	3	42	42.2
SZTDM-2_Bin289	2.85	89.2	0.0	109	57.1	45,963	3	33	42.3

^a^Genome completeness, contamination and strain heterogeneity were estimated using CheckM (Parks et al., 2015). The calculated heterogeneity values are all equal to 0.

*Comp.* completeness, *Cont.* contamination, *OGT* optimal growth temperature.

**Fig. 1:**
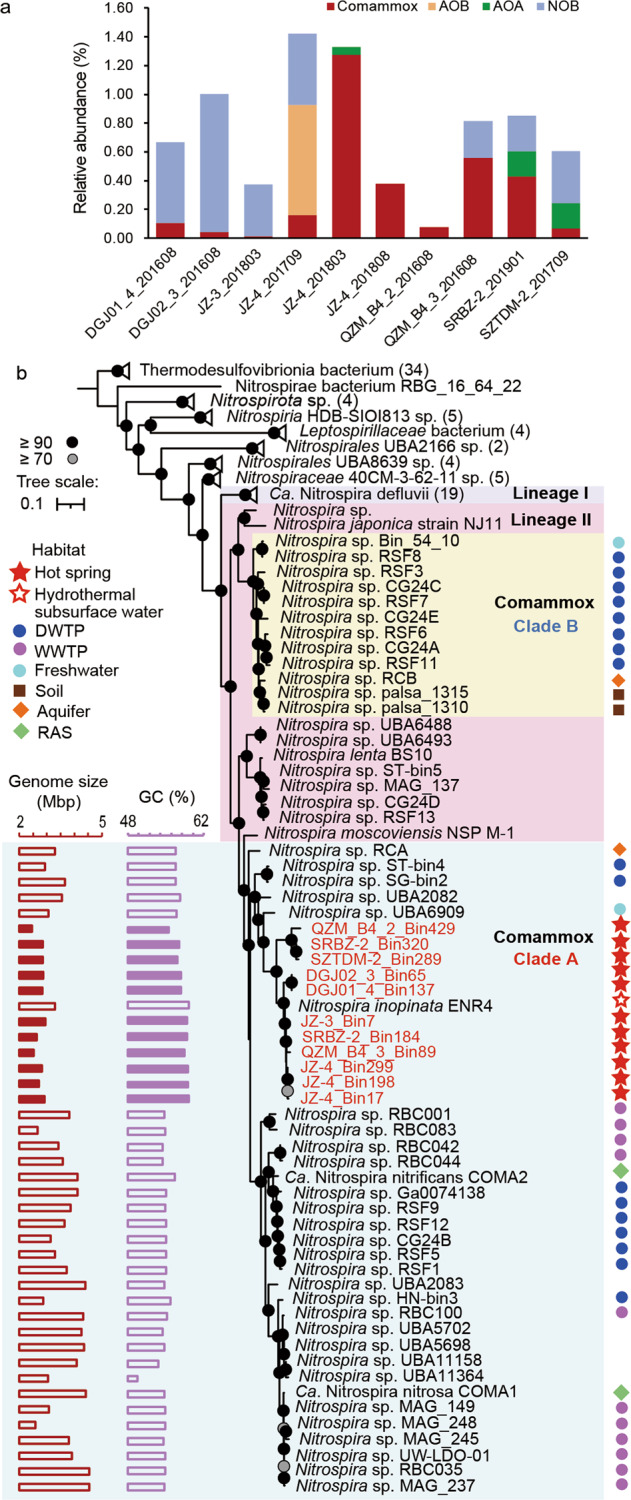
Overview of nitrifying communities in analyzed hot springs and phylogeny of hot spring comammox *Nitrospira*. **a** Inferred relative abundances of comammox *Nitrospira* and AOB MAGs in analyzed hot springs. **b** Phylogenomic affiliations of comammox *Nitrospira* MAGs in relation to selected reference organisms. The maximum likelihood tree was constructed using 120 marker proteins from in total 142 genomes (see Methods). Genomes from Thermotogae were used as the outgroup. *Nitrospira* lineage I and lineage II are highlighted by blue and purple boxes, respectively. Numbers in brackets at collapsed lineages indicate the numbers of genomes/MAGs per group. Comammox *Nitrospira* clade A and clade B are indicated by green and yellow backgrounds, respectively. The identifiers of comammox *Nitrospira* MAGs obtained in the present study are shown in red. Bootstrap values (based on 1000 iterations) ≥70 and ≥90 are indicated by gray and black circles, respectively. Genome size and GC content of each *Nitrospira* genome are given on the left side with MAGs from this study indicated by filled bars. The source habitats of genomes/MAGs are summarized on the right side of the tree: DWTP, drinking water treatment plant; WWTP, wastewater treatment plant; RAS, recirculating aquaculture system.

In these hot spring environments, comammox *Nitrospira* MAGs represented the dominant ammonia-oxidizing organisms (0.01 to 1.28% relative abundance compared to all bacteria and archaea detected in metagenomes) in eight out of ten samples, except for the samples JZ-4_201709 (38.0 °C) and SZTDM-2_201709 (54.0 °C), in which AOB and AOA showed a higher relative abundance, respectively (Fig. 1a). Additionally, NOB were also found present in seven samples at relative abundances of 0.24–0.96%. The phylogenomic analysis revealed that the 11 comammox MAGs formed two sub-clades within the comammox *Nitrospira* clade A (Fig. 1b), with eight MAGs in one cluster with *N. inopinata* and the remaining three forming an adjacent sister lineage. This clustering with *N. inopinata* showed that all currently known thermophilic strains within the clade A comammox *Nitrospira* are closely related to each other. These results indicate that the thermal tolerance of hot spring comammox *Nitrospira* is likely inherited from their last common ancestor within clade A, which acquired the thermal tolerance after moving into thermal habitats. However, one cannot rule out the existence of other thermophilic comammox strains that are divergent from this cluster.

### Predicted metabolic capacity of hot spring comammox *Nitrospira*

**Fig. 2:**
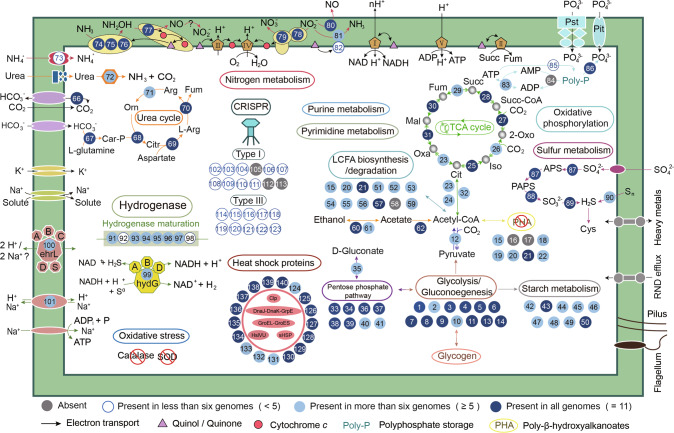
Cell cartoon based on the genome annotations of 11 hot-spring comammox *Nitrospira* MAGs. Shown are pathways and genes involved in metabolisms of carbon, hydrogen, nitrogen and sulfur, as well as respiration, electron transport, and stress defense. Metabolic modules are indicated by distinct color schemes. The presence of metabolism types across 11 hot spring comammox *Nitrospira* MAGs from this study is indicated as follows: dark blue solid circles represent all 11 MAGs; light blue solid circles represent 5 to 10 MAGs; light blue hollow circles represent 1 to 4 MAGs; and gray solid circles represent 0 MAGs. The numbering scheme associated with the shown pathways is given in Supplementary Dataset 2. Respiratory chain complexes are labelled by Roman numerals. Abbreviations: 2-Oxo 2-Oxo-glutarate, APS adenosine 5′-phosphosulfate, Arg arginine, Car-P carbamoyl-P, Cit citrate, Citr citrulline, Cys cysteine, EhrL energy-converting hydrogenase-related large subunit, Fum fumarate, HydG [NiFe]-Group 3b hydrogenases, Iso isocitrate, L-Arg L-Arginosuccinate, LCFA long-chain fatty acids, Mal malate, Orn ornithine, Oxa oxaloacetate, PAPS 3′-phosphoadenosine 5′ -phosphosulfate, PHA polyhydroxyalkanoate, Pit inorganic phosphate transporter, Poly-P polyphosphate, Pst phosphate transporter, rTCA cycle, reductive tricarboxylic acid cycle, sHSP small heat-shock protein, SOD superoxide dismutase, Suc succinate, Suc-CoA succinyl-CoA.

Consistent with their phylogenetic position within comammox *Nitrospira* clade A and confirming a comammox-type lifestyle, the 11 hot spring MAGs were found to contain genes of ammonia monooxygenase (*amoCAB*), hydroxylamine dehydrogenase (*hao*), and nitrite oxidoreductase (*nxrAB*), which are required for complete oxidation of ammonia to nitrate (Fig. 2). *AmoCAB* and *nxrAB* in each MAG co-localized on contiguous scaffolds and formed monophyletic clades with *N. inopinata* in phylogenetic analyses (Supplementary Fig. S1). While acknowledging the limitation given by incomplete MAGs, there is a notable absence of ammonia transporters (Rh50 and *amt*) in nearly all hot spring comammox *Nitrospira* MAGs, except for JZ-3_Bin7, despite their presumable dependency on ammonia for assimilatory processes. Despite undetected, urea is a general intracellular metabolite of prokaryotes and eukaryotes and is also released during the remineralization of organic matter [49]. A full complement of genes for urea utilization was observed in all 11 hot spring comammox *Nitrospira* MAGs (Fig. 2, Supplementary Fig. S2) [1, 4, 6], and the ammonia generated from intracellular urea hydrolysis may potentially support the growth of hot spring comammox *Nitrospira* in ammonium-limited environments. Analogous to other reported comammox genomes, hot spring comammox *Nitrospira* also lack an assimilatory nitrite reductase, suggesting their inability to grow on nitrite or nitrate as the only nitrogen source.

In comparison to mesophilic comammox *Nitrospira*, the pentaheme cytochrome *c* nitrite reductase (*nrfAH*) appears to be exclusively present in hot spring comammox MAGs (Fig. 2 and Supplementary Dataset 2) and the moderately thermophilic *N. inopinata* [1]. This suggests the potential of thermophilic comammox organisms to perform dissimilatory nitrate reduction to ammonium (DNRA), using the potentially reversible NXR for the initial reduction of nitrate to nitrite. This unique trait of comammox genomes recovered from high-temperature environments indicates a possible role of DNRA in their survival as previously suggested for other thermophilic bacteria and archaea [50, 51]. Specifically, the capability to perform DNRA provides an alternative mechanism for comammox *Nitrospira* to conserve energy under anoxic conditions, potentially allowing them also to survive in anoxic environments. Alternatively, using DNRA to produce ammonia could be a strategy for hot spring comammox *Nitrospira* to survive with nitrite as the only nitrogen source [1], and thus under the ammonium-limited conditions observed in hot springs. Even then, however, a low-potential electron donor for quinone reduction would be required to initiate the DNRA process and to provide electrons for biosynthetic processes, including CO_2_ fixation. In these pristine hot springs, organic metabolites (released by other organisms) or H_2_ (a potential product of the geochemical reduction of H_2_O at high temperatures and biomass fermentation at moderately thermal conditions) could be potential electron donors for DNRA. This is supported by the presence of key metabolic genes in the hot spring comammox *Nitrospira* MAGs that are potentially associated with these processes, i.e., *fadD*, *crt*, and *acd* for long-chain fatty acid catabolism, and the group 3b [NiFe] hydrogenase that might facilitate H_2_ oxidation (Fig. 2 and Supplementary Dataset 2). Together, these results suggest that hot spring comammox *Nitrospira* have versatile ecological functions as both ammonia sinks and sources.

**Fig. 3:**
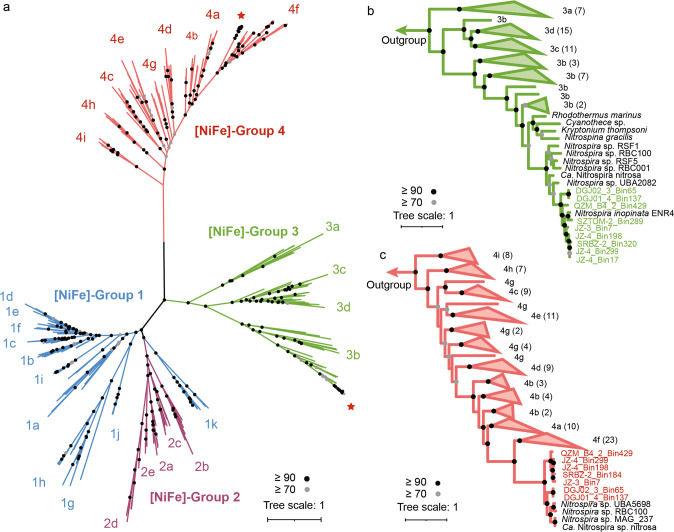
Classification and phylogeny of hydrogenases and energy-converting hydrogenase-related (Ehr) complexes. **a** The maximum likelihood tree shows the phylogenetic affiliation of hydrogenases and Ehr complexes identified in comammox *Nitrospira* in relation to 305 reference sequences. The tree is color-coded by hydrogenase subgroup. The red stars represent hydrogenases and Ehr complexes from hot spring comammox *Nitrospira*. The maximum likelihood trees show the phylogenetic affiliation of the **b** Group 3 [NiFe] hydrogenases and **c** Group 4 [NiFe] hydrogenases and Ehr complexes identified in comammox *Nitrospira* and reference hydrogenases. Bootstrap values (based on 1000 iterations) ≥ 70 and ≥ 90 are indicated by gray and black circles, respectively.

In high-temperature environments, metabolic strategies that utilize or produce H_2_ represent an essential approach to energy conservation by chemolithoautotrophic microorganisms [23, 52]. The bidirectional group 3b [NiFe] hydrogenases and accessory proteins for hydrogenase maturation, which widely occur in canonical NOB and comammox *Nitrospira* [1, 53, 54], have been also observed in nearly all hot spring comammox *Nitrospira* (Fig. 3a, b). As observed in a hyperthermophilic archaeon (*Pyrococcus furiosus*) [55], the group 3b [NiFe] hydrogenases might also participate in sulfur cycling, which is another key element commonly found in terrestrial hot springs. Despite these speculated roles, the bona fide function of the group 3b [NiFe] hydrogenases in *Nitrospira* has not been characterized thus far. Moreover, we also observed the presence of energy-converting hydrogenase-related complexes (Ehr) in seven hot spring comammox *Nitrospira* MAGs presented here (Fig. 3c and Supplementary Fig. S3), which are phylogenetically close to the group 4 f [NiFe] hydrogenases but lack the typical CxxC motifs required for binding of the [NiFe] center. Thus, these Ehr complexes unlikely perform as [NiFe] hydrogenases and their function remains unknown. However, it is interesting to note their similarity to subunits of respiratory Complex I and of energy-converting hydrogenases (Ech), which are involved in electron transport and energy transduction [56].

### Transcription of core metabolic pathways

**Fig. 4:**
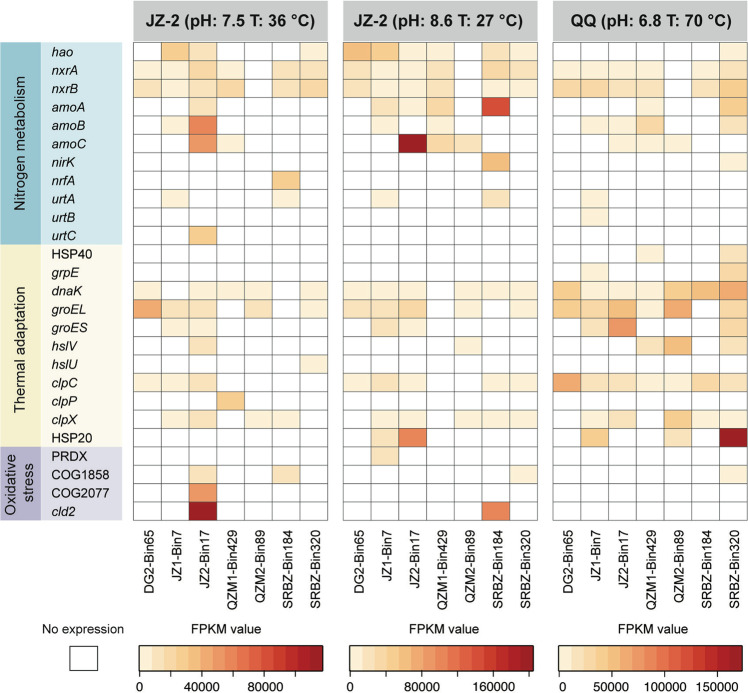
Gene expression of seven comammox *Nitrospira* MAGs for nitrogen metabolism, thermal adaptation and oxidative stress across three hot springs derived metatranscriptomes. Colour intensity represents relative abundance of gene expression as measured by FPKM (Methods section). The white boxes indicate the gene expression is undetectable.

The in situ activity of hot spring comammox *Nitrospira* was affirmed by metatranscriptomic sequencing (Fig. 4). To avoid biases in estimating gene expression levels of highly similar MAGs, we manually selected seven out of the 11 hot spring comammox MAGs as reference genomes based on an AAI ≥ 95% and genome quality (Supplementary Table S2). From this analysis, transcriptional activities of 976 out of 19142 (5.1%) encoded genes in these seven MAGs were detected, suggesting that these organisms have an active role in the three hot spring samples that were measured.

The nitrification pathway was at least partially expressed across all seven hot spring comammox reference MAGs in all three metatranscriptomes (Fig. 4). For example, in JZ-2, JZ-4, and QQ, the MAGs JZ-4_Bin17, QZM-B4_2_Bin429, and SRBZ-2_Bin320, respectively, expressed the entire suite of genes related to nitrification including *amoCAB*, *hao*, and *nxrAB*. Moreover, the expression of nitrification-related genes accounted for the majority of transcripts assigned to the hot spring comammox *Nitrospira* MAGs. The expression of genes associated with nitrification constituted 4.4% and 4.0% of comammox transcripts for SBRZ-2_Bin320 in sample QQ and JZ-4_Bin17 in sample JZ-2, respectively (Supplementary Fig. S4). This indicates that nitrification was a key process catalyzed by comammox *Nitrospira* in the examined hot springs. Additionally, low expression levels of other nitrogen metabolism pathways were observed in at least one hot spring comammox *Nitrospira* MAG. These genes included NO-forming nitrite reductase (*nirK*), pentaheme cytochrome c nitrite reductase (*nrfA*), and urea transporters (*urtABC*). This suggests that hot spring comammox *Nitrospira* can be involved in additional nitrogen-cycling processes such as nitrite reduction to nitric oxide, DNRA, and urea utilization.

Moreover, the expression of complete nitrification pathway was not consistently observed for all comammox MAGs. For example, DGJ02_3_Bin65 expressed *nxrAB* in all three samples while no *hao* or *amoCAB* transcripts were detected, and *amoC* appeared to be the only transcribed nitrification-related genes by QZM_B4_3_Bin89. This lack of nitrification gene expression by some comammox strains might reflect limited metabolic activity of these organisms under extreme conditions in hot springs. Similar to our observations, partial expression of nitrification-related genes in comammox *Nitrospira* was also observed in a previous study [57], where the transcripts of *amoCAB* and *nxrAB* were inconsistently detected across different comammox species.

The transcription of genes associated with thermal adaptation also confirmed the activity of hot spring comammox *Nitrospira* and shed light on their potential survival mechanisms. Heat shock proteins (HSPs) are expressed by cells in response to external stress. They contribute to removing and refolding damaged proteins, protein assembly, secretion, and regulation of transcription factors, and may also play a protective role for other proteins by forming temperature-dependent complexes [58]. As expected, a variety of chaperones were encoded and transcribed by all seven selected hot spring comammox *Nitrospira* MAGs (Fig. 4 and Supplementary Dataset 2). For example, the gene for the highly conserved chaperone protein DnaK, which functions in protein folding and preventing the aggregation of unfolding proteins under stress [59], was expressed by nearly all hot spring comammox *Nitrospira* MAGs. Several copies of small HSPs (HSP20) were detected in SRBZ2-Bin320 and exhibited high expression levels in the metatranscriptomic dataset from the sample QQ (Supplementary Fig. S4). Chaperonins of the GroEL-GroES and caseinolytic protease (Clp) families were also highly transcribed by hot spring comammox *Nitrospira*. GroEL-GroES chaperonins can encapsulate and fold proteins, while Clp family members may regulate the disaggregation and degradation of distorted proteins and are also able to orchestrate DnaK to refold misfolded proteins [60]. Additionally, nucleotide-exchange factor (GrpE), ATP-dependent heat-shock locus genes (HslVU), and several genes associated with oxidative stress defense were highly expressed, indicating that diverse thermal stress mitigation mechanisms are adopted by comammox *Nitrospira*.

### Unique amino acid composition of hot spring comammox *Nitrospira*

Adjusting the amino acid compositions of proteins is an important adaptation strategy of microorganisms to overcome thermal stress [61, 62]. In agreement with this hypothesis, comparing the amino acid compositions of 30 clade A comammox *Nitrospira* MAGs (Supplementary Text S1) revealed that comammox *Nitrospira* recovered from thermal and non-thermal habitats have evolved distinct amino acid usage patterns (Supplementary Fig. S5). Specifically, thermophilic comammox *Nitrospira* utilize more non-polar and charged amino acids at the expense of polar amino acids (*p* < 0.01). This phenomenon may be attributed to the chemical characteristics of different amino acids: strong ionic bonds between oppositely charged amino acid residues increase the stability of a protein, while polar amino acids (e.g., Asn and Gln) generally result in a decrease in protein stability due to thermal deamidation [63, 64]. The specific amino acid usage pattern by thermophilic comammox *Nitrospira* is in line with a previous comparison between the amino acid composition of 60 thermophilic proteins and their mesophilic homologues [65], as well as general observations on protein composition in other thermophiles [64, 66]. These results strongly suggest that thermophilic phenotypes of comammox *Nitrospira* can be identified based on their predicted amino acid composition deduced from genomic materials. Consequently, we further tested a typical amino acid composition-based index, the IVYWREL index [67], to decern thermophilic comammox *Nitrospira* from non-thermophilic members. As expected, the IVYWREL index was significantly different (*p* < 0.01) for thermophilic and mesophilic comammox *Nitrospira* (Supplementary Fig. S6), providing further evidence for compositional differences based on their amino acid usage profiles. Notably, the IVYWREL index of *Nitrospira* sp. ST-BIN4, *Nitrospira* sp. SG-bin2, *Nitrospira* sp. UBA2082, and *Nitrospira* sp. UBA6909, which were obtained from non-thermal habitats but are phylogenetically close to thermal comammox *Nitrospira* (Fig. 1), was determined to be 40.0~40.3 and thus also significantly lower than that of thermal comammox *Nitrospira* (*p* < 0.01). This lends strong support to the hypothesis that the observed unique amino acid profiles are genuinely due to the thermophilic phenotype rather than phylogenetic affiliation.

### Genomic properties shed light on the adaptation of hot spring comammox *Nitrospira*

**Fig. 5:**
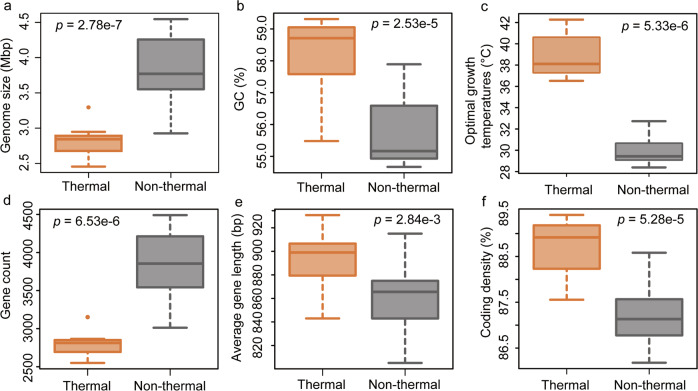
Comparisons of general genomic properties between 12 thermal and 18 non-thermal clade A comammox *Nitrospira* (Supplementary Dataset 4). Statistical analysis was conducted using the Wilcoxon rank sum test. **a** genome size; **b** GC content; **c** predicted optimal growth temperature (OGT); **d** gene count; **e** average gene length; **f** coding density.

In addition to the changes in amino acid utilization, we also compared the genomic properties of 30 clade A comammox *Nitrospira* MAGs (Supplementary Text S1) and observed several notable differences between thermal and non-thermal clade A comammox *Nitrospira* that may shed light on possible adaptation strategies in high-temperature environments. Specifically, the thermophilic comammox *Nitrospira* MAGs have a significantly smaller genome size (2.46–3.30 Mbp) than mesophilic clade A comammox *Nitrospira* (2.93–4.55 Mbp; *p* < 0.05) (Fig. 5a). This is not surprising, as genome size reduction is considered to be beneficial for microbial heat tolerance through the reduced energy requirements for nucleotide synthesis [68]. For example, Sabath et al. [68] compared more than 1000 prokaryotes genomes and observed significant differences in the genome sizes between mesophiles, thermophiles, and hyperthermophiles, and Sorensen et al. [69] reported a significant and negative correlation between the average genome size and the site temperature in 12 metagenomes obtained from fire-affected soils. Correspondingly, a strong correlation between the genome size and the predicted optimal growth temperature (OGT, see details in Supplementary Text S1) was observed here for the 30 clade A comammox *Nitrospira* genomes (Supplementary Fig. S7).

Another important genomic feature is the GC content. With contradictory results observed in different studies [67, 70, 71], the correlation between GC content and microbial tolerance against heat stress is still elusive. Here, we found that the GC content was significantly higher in thermophilic comammox *Nitrospira* MAGs than in non-thermal clade A comammox *Nitrospira* (55.48–59.31% vs. 54.67–57.89%; *p* < 0.05) (Fig. 5b). As expected, the OGT was also found to be significantly higher for the genomes derived from thermal habitats (Fig. 5c, p < 0.05). Additionally, the observed decreased gene count, increased average gene length, and increased coding density (Fig. 5d-f) indicate that dispensable and low-stability proteins might have been preferentially lost from the genomes of these thermophilic comammox *Nitrospira* [72]. Moreover, thermophilic comammox *Nitrospira* generally possess fewer genes encoding for transporters and bacterial secretion systems in comparison to non-thermal clade A comammox *Nitrospira* (Supplementary Fig. S8). This observation is in agreement with the hypothesis that the reduction of functional complexity is likely a cost-minimizing and energy-saving mechanism for thermophiles to adapt to elevated temperatures [73]. Taken together, the observed genomic features of thermophilic comammox *Nitrospira* indicate that these organisms are well adapted to survive in high-temperature ecosystems.

### Ecological implications

The discovery and characterization of comammox *Nitrospira* significantly changed our understanding of the microbially mediated nitrogen cycle, as it overturned a century of accumulated knowledge by demonstrating that complete nitrification can be carried out by a single rather than multiple microorganisms in concert. Here, we report the detection of comammox *Nitrospira* in hot spring environments at temperatures up to 80 °C and their transcriptional activities at temperatures up to 70 °C. While numerous efforts have been made to show the ubiquitous existence of comammox *Nitrospira* in different environmental settings [12, 13, 53], the present study adds a key element to research into biogeochemical nitrogen cycling in high-temperature environments.

In comparison to human-affected (e.g., contaminated, fertilized, or engineered) soils and aquatic systems, the ammonium concentrations in the examined hot springs were much lower. This could theoretically explain the dominance of comammox *Nitrospira* over the other ammonia-oxidizing guilds in eight out of ten samples because they likely are well adapted to nitrogen-limited conditions [4, 5]. Additionally, the comparison of genomic properties showed relatively smaller genome sizes of hot spring comammox bacteria, which generally reflects a smaller cell size [74, 75] and tends to be advantageous for optimizing the surface-to-volume ratio for the optimal uptake of low-abundance nutrients [76–78]. However, limited ammonia availability may not be the only factor favoring their occurrence, as comammox *Nitrospira* were found to dominate over other ammonia oxidizers in many soil systems where the ammonium concentration varied from 0.1 to 20 mg kg^−1^ soil [79–82]. Other environmental factors such as pH, oxygen level, temperature, and salinity also likely contribute to the niche differentiation among ammonia oxidizers [79, 81], and their differential responses to these factors are often used to explain their co-occurrence, or to compare interspecies competitiveness of different ammonia-oxidizing guilds. Temperature and pH directly influence the kinetics of ammonia oxidizers [78, 83, 84], and a recent study demonstrated that the ammonia affinity constants of *N. inopinata* and the AOA ‘*Candidatus* Nitrosocosmicus oleophilus MY3’ increased 3–4 times when the pH was elevated from ~6.5 to ~8.5 [78].

We also searched the 16S rRNA and *amoA* gene sequences retrieved from SRBZ-2_Bin320 against the public database and found highly similar sequences in other thermal habitats in Russia, India, Argentina, and Australia (Supplementary Text S2 and Dataset 3), suggesting a potentially wide distribution of comammox *Nitrospira* in thermal habitats. It should be noted that these detected strains identified based on short sequences are only potential comammox bacteria. Future studies, including cultivation efforts in combination with metagenomic, metatranscriptomic, and metaproteomic analyses, are required to confirm their roles in ammonia oxidation in these systems.

## Conclusions

Collectively, this study confirms the frequent distribution and transcriptional activity of comammox *Nitrospira* in high-temperature ecosystems and highlights their contributions to nitrogen conversions therein. By comparing thermal and non-thermal comammox bacteria, the results provide insights into potential adaptation strategies that hot spring comammox bacteria use against heat stress, including adapted amino acid usage profiles, modified genomic features, and expression of a large number of HSPs. This study is also of particular importance as terrestrial hot springs represent an important niche supporting a variety of chemolithoautotrophic extremophiles, and understanding the microbial nitrogen conversions therein is highly relevant from the ecological point of view [19–21]. While the contributions of AOB and AOA to nitrogen cycling in hot springs have been documented previously [24–27, 29, 85], the present findings fill an important knowledge gap by providing evidence for the occurrence and activities of comammox bacteria in the examined hot springs.

While the in situ transcriptomic evidence presented here confirms the activity of comammox *Nitrospira* in hot springs for the first time, follow-up research will be needed to further validate the results. Once enrichment cultures of these comammox organisms become available, targeted physiological experiments can provide more direct evidence of their activities, thermal adaptations, and further ecophysiological characteristics. Currently, environmental data, for example the concentrations of H_2_, organic compounds, urea, and dissolved oxygen are lacking for most hot springs we analyzed. Such information will be needed to better understand how thermophilic nitrifiers, including comammox bacteria, cope with extreme conditions and regulate their metabolism in a variable environment.

## Supplementary information

SUPPLEMENTAL MATERIAL

Supplementary Dataset 1

Supplementary Dataset 2

Supplementary Dataset 3

Supplementary Dataset 4

## Data Availability

The genome bins used in this study have been deposited at NCBI under the project of PRJNA851927.

## References

[1] Daims H, Lebedeva EV, Pjevac P, Han P, Herbold C, Albertsen M (2015). Complete nitrification by *Nitrospira* bacteria. Nature.

[2] van Kessel MA, Speth DR, Albertsen M, Nielsen PH, den Camp HJO, Kartal B (2015). Complete nitrification by a single microorganism. Nature.

[3] Koch H, Lücker S, Albertsen M, Kitzinger K, Herbold C, Spieck E (2015). Expanded metabolic versatility of ubiquitous nitrite-oxidizing bacteria from the genus *Nitrospira*. Proc Natl Acad Sci USA.

[4] Kits KD, Sedlacek CJ, Lebedeva EV, Han P, Bulaev A, Pjevac P (2017). Kinetic analysis of a complete nitrifier reveals an oligotrophic lifestyle. Nature.

[5] Sakoula D, Koch H, Frank J, Jetten MS, van Kessel MA, Lücker S (2021). Enrichment and physiological characterization of a novel comammox *Nitrospira* indicates ammonium inhibition of complete nitrification. ISME J.

[6] Li J, Hua Z-S, Liu T, Wang C, Li J, Bai G (2021). Selective enrichment and metagenomic analysis of three novel comammox *Nitrospira* in a urine-fed membrane bioreactor. ISME commun.

[7] Wang Y, Zhao R, Liu L, Li B, Zhang T (2021). Selective enrichment of comammox from activated sludge using antibiotics. Water Res.

[8] Shi X, Hu H-W, Wang J, He J-Z, Zheng C, Wan X (2018). Niche separation of comammox *Nitrospira* and canonical ammonia oxidizers in an acidic subtropical forest soil under long-term nitrogen deposition. Soil Biol Biochem.

[9] Sun D, Tang X, Zhao M, Zhang Z, Hou L, Liu M (2020). Distribution and diversity of comammox *Nitrospira* in coastal wetlands of China. Front Microbiol.

[10] Pjevac P, Schauberger C, Poghosyan L, Herbold CW, Van Kessel MA, Daebeler A (2017). *AmoA*-targeted polymerase chain reaction primers for the specific detection and quantification of comammox *Nitrospira* in the environment. Front Microbiol.

[11] Pinto AJ, Marcus DN, Ijaz UZ, Bautista-de lose Santos QM, Dick GJ, Raskin L. Metagenomic evidence for the presence of comammox *Nitrospira*-like bacteria in a drinking water system. *mSphere*. 2016;1:e00054–00015. 10.1128/mSphere.00054-15 PMC4863621 27303675

[12] Fowler SJ, Palomo A, Dechesne A, Mines PD, Smets BF (2018). Comammox *Nitrospira* are abundant ammonia oxidizers in diverse groundwater‐fed rapid sand filter communities. Environ Microbiol.

[13] Palomo A, Fowler SJ, Gülay A, Rasmussen S, Sicheritz-Ponten T, Smets BF. Metagenomic analysis of rapid gravity sand filter microbial communities suggests novel physiology of *Nitrospira* spp. *ISME J*. 2016;10:2569–81. 10.1038/ismej.2016.63 PMC5113852 27128989

[14] Cotto I, Dai Z, Huo L, Anderson CL, Vilardi KJ, Ijaz U (2020). Long solids retention times and attached growth phase favor prevalence of comammox bacteria in nitrogen removal systems. Water Res.

[15] Spasov E, Tsuji JM, Hug LA, Doxey AC, Sauder LA, Parker WJ, et al. High functional diversity among *Nitrospira* populations that dominate rotating biological contactor microbial communities in a municipal wastewater treatment plant. *ISME J*. 2020;14:1857–72. 10.1038/s41396-020-0650-2 PMC7305129 32332864

[16] Yang Y, Daims H, Liu Y, Herbold CW, Pjevac P, Lin J-G (2020). Activity and metabolic versatility of complete ammonia oxidizers in full-scale wastewater treatment systems. Mbio.

[17] Zhao J, Zheng M, Su Z, Liu T, Li J, Guo J (2022). Selective enrichment of comammox *Nitrospira* in a moving bed biofilm reactor with sufficient oxygen supply. Environ Sci Technol.

[18] Huang T, Xia J, Liu T, Su Z, Guan Y, Guo J (2022). Comammox *Nitrospira* bacteria are dominant ammonia oxidizers in mainstream nitrification bioreactors emended with sponge carriers. Environ Sci Technol.

[19] Williams TA, Szöllősi GJ, Spang A, Foster PG, Heaps SE, Boussau B (2017). Integrative modeling of gene and genome evolution roots the archaeal tree of life. Proc Natl Acad Sci USA.

[20] Djokic T, Van Kranendonk MJ, Campbell KA, Walter MR, Ward CR (2017). Earliest signs of life on land preserved in ca. 3.5 Ga hot spring deposits. Nat Commun.

[21] Damer B, Deamer D (2020). The hot spring hypothesis for an origin of life. Astrobiology.

[22] Davis BM (1897). The vegetation of the hot springs of Yellowstone Park. Science.

[23] Reysenbach A-L, Shock E (2002). Merging genomes with geochemistry in hydrothermal ecosystems. Science.

[24] Zhang CL, Ye Q, Huang Z, Li W, Chen J, Song Z (2008). Global occurrence of archaeal *amoA* genes in terrestrial hot springs. Appl Environ Microbiol.

[25] Daebeler A, Herbold CW, Vierheilig J, Sedlacek CJ, Pjevac P, Albertsen M (2018). Cultivation and genomic analysis of “*Candidatus* Nitrosocaldus islandicus,” an obligately thermophilic, ammonia-oxidizing thaumarchaeon from a hot spring biofilm in Graendalur Valley, Iceland. Front Microbiol.

[26] Reigstad LJ, Richter A, Daims H, Urich T, Schwark L, Schleper C (2008). Nitrification in terrestrial hot springs of Iceland and Kamchatka. FEMS Microbiol Ecol.

[27] Hatzenpichler R, Lebedeva EV, Spieck E, Stoecker K, Richter A, Daims H (2008). A moderately thermophilic ammonia-oxidizing crenarchaeote from a hot spring. Proc Natl Acad Sci USA.

[28] Edwards TA, Calica NA, Huang DA, Manoharan N, Hou W, Huang L (2013). Cultivation and characterization of thermophilic *Nitrospira* species from geothermal springs in the US Great Basin, China, and Armenia. FEMS Microbiol Ecol.

[29] Lebedeva EV, Alawi M, Fiencke C, Namsaraev B, Bock E, Spieck E (2005). Moderately thermophilic nitrifying bacteria from a hot spring of the Baikal rift zone. FEMS Microbiol Ecol.

[30] Spieck E, Spohn M, Wendt K, Bock E, Shively J, Frank J (2020). Extremophilic nitrite-oxidizing *Chloroflexi* from Yellowstone hot springs. ISME J.

[31] Hua Z-S, Wang Y-L, Evans PN, Qu Y-N, Goh KM, Rao Y-Z (2019). Insights into the ecological roles and evolution of methyl-coenzyme M reductase-containing hot spring Archaea. Nat Commun.

[32] Hua Z-S, Han Y-J, Chen L-X, Liu J, Hu M, Li S-J (2015). Ecological roles of dominant and rare prokaryotes in acid mine drainage revealed by metagenomics and metatranscriptomics. ISME J.

[33] Bankevich A, Nurk S, Antipov D, Gurevich AA, Dvorkin M, Kulikov AS (2012). SPAdes: a new genome assembly algorithm and its applications to single-cell sequencing. J Comput Biol.

[34] Kang DD, Froula J, Egan R, Wang Z (2015). MetaBAT, an efficient tool for accurately reconstructing single genomes from complex microbial communities. PeerJ.

[35] Dick GJ, Andersson AF, Baker BJ, Simmons SL, Thomas BC, Yelton AP (2009). Community-wide analysis of microbial genome sequence signatures. Genome Biol.

[36] Parks DH, Imelfort M, Skennerton CT, Hugenholtz P, Tyson GW (2015). CheckM: assessing the quality of microbial genomes recovered from isolates, single cells, and metagenomes. Genome Res.

[37] Chaumeil P-A, Mussig AJ, Hugenholtz P, Parks DH (2019). GTDB-Tk: a toolkit to classify genomes with the Genome Taxonomy Database. Bioinformatics.

[38] Hua Z-S, Qu Y-N, Zhu Q, Zhou E-M, Qi Y-L, Yin Y-R (2018). Genomic inference of the metabolism and evolution of the archaeal phylum Aigarchaeota. Nat Commun.

[39] Hyatt D, Chen G-L, LoCascio PF, Land ML, Larimer FW, Hauser LJ (2010). Prodigal: prokaryotic gene recognition and translation initiation site identification. BMC Bioinform.

[40] Buchfink B, Xie C, Huson DH (2015). Fast and sensitive protein alignment using DIAMOND. Nat Methods.

[41] Brazelton WJ, Nelson B, Schrenk MO (2012). Metagenomic evidence for H_2_ oxidation and H_2_ production by serpentinite-hosted subsurface microbial communities. Front Microbiol.

[42] Edgar RC (2004). MUSCLE: multiple sequence alignment with high accuracy and high throughput. Nucleic Acids Res.

[43] Capella-Gutiérrez S, Silla-Martínez JM, Gabaldón T (2009). trimAl: a tool for automated alignment trimming in large-scale phylogenetic analyses. Bioinformatics.

[44] Nguyen L-T, Schmidt HA, Von Haeseler A, Minh BQ (2015). IQ-TREE: a fast and effective stochastic algorithm for estimating maximum-likelihood phylogenies. Mol Biol Evol.

[45] Kalyaanamoorthy S, Minh BQ, Wong TK, Von Haeseler A, Jermiin LS (2017). ModelFinder: fast model selection for accurate phylogenetic estimates. Nat Methods.

[46] Li H, Handsaker B, Wysoker A, Fennell T, Ruan J, Homer N (2009). The sequence alignment/map format and SAMtools. Bioinformatics.

[47] Quinlan AR, Hall IM (2010). BEDTools: a flexible suite of utilities for comparing genomic features. Bioinformatics.

[48] Trapnell C, Williams BA, Pertea G, Mortazavi A, Kwan G, Van Baren MJ (2010). Transcript assembly and quantification by RNA-Seq reveals unannotated transcripts and isoform switching during cell differentiation. Nat Biotechnol.

[49] Kitzinger K, Padilla CC, Marchant HK, Hach PF, Herbold CW, Kidane AT (2019). Cyanate and urea are substrates for nitrification by Thaumarchaeota in the marine environment. Nat Microbiol.

[50] Dodsworth JA, Hungate BA, Hedlund BP (2011). Ammonia oxidation, denitrification and dissimilatory nitrate reduction to ammonium in two US Great Basin hot springs with abundant ammonia‐oxidizing archaea. Environ Microbiol.

[51] Vetriani C, Speck MD, Ellor SV, Lutz RA, Starovoytov V (2004). *Thermovibrio ammonificans* sp. nov., a thermophilic, chemolithotrophic, nitrate-ammonifying bacterium from deep-sea hydrothermal vents. Int J Syst Evol Microbiol.

[52] Kim YJ, Lee HS, Kim ES, Bae SS, Lim JK, Matsumi R (2010). Formate-driven growth coupled with H_2_ production. Nature.

[53] Poghosyan L, Koch H, Lavy A, Frank J, van Kessel MA, Jetten MS (2019). Metagenomic recovery of two distinct comammox *Nitrospira* from the terrestrial subsurface. Environ Microbiol.

[54] Lücker S, Nowka B, Rattei T, Spieck E, Daims H (2013). The genome of *Nitrospina gracilis* illuminates the metabolism and evolution of the major marine nitrite oxidizer. Front Microbiol.

[55] Ma K, Weiss R, Adams MW (2000). Characterization of hydrogenase II from the hyperthermophilic archaeon *Pyrococcus furiosus* and assessment of its role in sulfur reduction. J Bacteriol.

[56] Marreiros BC, Batista AP, Duarte AM, Pereira MM (2013). A missing link between complex I and group 4 membrane-bound [NiFe] hydrogenases. Biochim Biophys Acta.

[57] Liu S, Cai H, Wang J, Wang H, Zheng T, Chen Q (2021). In-situ expressions of comammox *Nitrospira* along the Yangtze River. Water Res.

[58] Feng L, Wang W, Cheng J, Ren Y, Zhao G, Gao C (2007). Genome and proteome of long-chain alkane degrading *Geobacillus thermodenitrificans* NG80-2 isolated from a deep-subsurface oil reservoir. Proc Natl Acad Sci USA.

[59] Kampinga HH, Craig EA (2010). The HSP70 chaperone machinery: J proteins as drivers of functional specificity. Nat Rev Mol Cell Biol.

[60] Richter K, Haslbeck M, Buchner J (2010). The heat shock response: life on the verge of death. Mol Cell.

[61] Wang Q, Cen Z, Zhao J (2015). The survival mechanisms of thermophiles at high temperatures: an angle of omics. Physiology.

[62] Zheng H, Wu H (2010). Gene-centric association analysis for the correlation between the guanine-cytosine content levels and temperature range conditions of prokaryotic species. BMC Bioinform.

[63] Fukuchi S, Nishikawa K (2001). Protein surface amino acid compositions distinctively differ between thermophilic and mesophilic bacteria. J Mol Biol.

[64] López-García P, Zivanovic Y, Deschamps P, Moreira D (2015). Bacterial gene import and mesophilic adaptation in archaea. Nat Rev Microbiol.

[65] Liang HK, Huang CM, Ko MT, Hwang JK (2005). Amino acid coupling patterns in thermophilic proteins. Proteins: Struct Funct Bioinform.

[66] Hickey DA, Singer GA (2004). Genomic and proteomic adaptations to growth at high temperature. Genome Biol.

[67] Zeldovich KB, Berezovsky IN, Shakhnovich EI (2007). Protein and DNA sequence determinants of thermophilic adaptation. PLoS Comput Biol.

[68] Sabath N, Ferrada E, Barve A, Wagner A (2013). Growth temperature and genome size in bacteria are negatively correlated, suggesting genomic streamlining during thermal adaptation. Genome Biol Evol.

[69] Sorensen JW, Dunivin TK, Tobin TC, Shade A (2019). Ecological selection for small microbial genomes along a temperate-to-thermal soil gradient. Nat Microbiol.

[70] Gu W, Zhou T, Wilke CO (2010). A universal trend of reduced mRNA stability near the translation-initiation site in prokaryotes and eukaryotes. PLoS Comput Biol.

[71] Wang H-C, Susko E, Roger AJ (2006). On the correlation between genomic G + C content and optimal growth temperature in prokaryotes: data quality and confounding factors. Biochem Biophys Res Co.

[72] Kuo C-H, Moran NA, Ochman H (2009). The consequences of genetic drift for bacterial genome complexity. Genome Res.

[73] Burra PV, Kalmar L, Tompa P (2010). Reduction in structural disorder and functional complexity in the thermal adaptation of prokaryotes. PloS One.

[74] Lynch M, Walsh B (2007). The origins of genome architecture. Vol. 98.

[75] Giovannoni SJ, Cameron Thrash J, Temperton B (2014). Implications of streamlining theory for microbial ecology. ISME J.

[76] Giovannoni SJ, Tripp HJ, Givan S, Podar M, Vergin KL, Baptista D (2005). Genome streamlining in a cosmopolitan oceanic bacterium. Science.

[77] Moya A, Gil R, Latorre A, Peretó J, Pilar Garcillán-Barcia M, De La Cruz F (2008). Toward minimal bacterial cells: evolution vs. design. FEMS Microbiol Rev.

[78] Jung M-Y, Sedlacek CJ, Kits KD, Mueller AJ, Rhee S-K, Hink L (2022). Ammonia-oxidizing archaea possess a wide range of cellular ammonia affinities. ISME J.

[79] He Z-Y, Shen J-P, Zhang L-M, Tian H-J, Han B, Di H-J (2020). DNA stable isotope probing revealed no incorporation of ^13^CO_2_ into comammox *Nitrospira* but ammonia-oxidizing archaea in a subtropical acid soil. J Soils Sediment.

[80] Liu T, Wang Z, Wang S, Zhao Y, Wright AL, Jiang X (2019). Responses of ammonia-oxidizers and comammox to different long-term fertilization regimes in a subtropical paddy soil. Eur J Soil Biol.

[81] Osburn ED, Barrett J (2020). Abundance and functional importance of complete ammonia-oxidizing bacteria (comammox) versus canonical nitrifiers in temperate forest soils. Soil Biol Biochem.

[82] Li C, He Z-Y, Hu H-W, He J-Z. Niche specialization of comammox *Nitrospira* in terrestrial ecosystems: Oligotrophic or copiotrophic? *Crit Rev Env Sci Technol*. 2023;53:161–76.

[83] Aigle A, Gubry‐Rangin C, Thion C, Estera‐Molina K, Richmond H, Pett‐Ridge J (2020). Experimental testing of hypotheses for temperature‐ and pH‐based niche specialization of ammonia oxidizing archaea and bacteria. Environ Microbiol.

[84] Groeneweg J, Sellner B, Tappe W (1994). Ammonia oxidation in *Nitrosomonas* at NH_3_ concentrations near K_m_: effects of pH and temperature. Water Res.

[85] Jaeschke A, Op den Camp HJ, Harhangi H, Klimiuk A, Hopmans EC, Jetten MS (2009). 16S rRNA gene and lipid biomarker evidence for anaerobic ammonium-oxidizing bacteria (anammox) in California and Nevada hot springs. FEMS Microbiol Ecol.

