# Supplementary material for: Hot spring distribution and survival mechanisms of thermophilic comammox Nitrospira
Source: ISME J. 2023 Apr 17;17(7):993–1003. doi: 10.1038/s41396-023-01409-w (PMC10284858; doi:10.1038/s41396-023-01409-w)
Supplement: Supplementary file 1 — SUPPLEMENTAL MATERIAL [file 41396_2023_1409_MOESM1_ESM.doc]

Supplementary information

**Hot spring distribution and survival mechanisms of thermophilic comammox *Nitrospira***

Yan Zhang^1,10^, Tao Liu^2,10^, Meng-Meng Li^3,10^, Zheng-Shuang Hua^4,*^, Paul Evans^5^, Yanni Qu^3^, Sha Tan^3^, Min Zheng^2^, Hui Lu^6^, Jian-Yu Jiao^3^, Sebastian Lücker^7^, Holger Daims^8,9^, Wen-Jun Li^3,*^, Jianhua Guo^2,*^

^1^School of Environmental and Chemical Engineering, Foshan University, Foshan, China

^2^Australian Centre for Water and Environmental Biotechnology, Faculty of Engineering, Architecture and Information Technology, The University of Queensland, St Lucia, Queensland, Australia

^3^State Key Laboratory of Biocontrol, Guangdong Provincial Key Laboratory of Plant Resources and Southern Marine Science and Engineering Guangdong Laboratory (Zhuhai), School of Life Sciences, Sun Yat-sen University, Guangzhou, China

^4^Department of Environmental Science and Engineering, University of Science and Technology of China, Hefei, China

^5^The Australian Centre for Ecogenomics, School of Chemistry and Molecular Biosciences, University of Queensland, St Lucia, Queensland, Australia

^6^School of Environmental Science and Engineering, Sun Yat-sen University, Guangzhou, 510275, China

^7^Department of Microbiology, RIBES, Radboud University, Heyendaalseweg 135, 6525 AJ, Nijmegen, the Netherlands

^8^Division of Microbial Ecology, Centre for Microbiology and Environmental Systems Science, University of Vienna, Djerassiplatz 1, 1030 Vienna, Austria

^9^The Comammox Research Platform, University of Vienna, Djerassiplatz 1, 1030 Vienna, Austria

^10^These authors contributed equally: Yan Zhang, Tao Liu, Meng-Meng Li

^*^Corresponding author email: [hzhengsh@ustc.edu.cn](mailto:hzhengsh@ustc.edu.cn); [liwenjun3@mail.sysu.edu.cn](mailto:liwenjun3@mail.sysu.edu.cn); [jianhua.guo@uq.edu.au](mailto:jianhua.guo@uq.edu.au).

**Table and Figure legends**

**Supplementary Table S1** Physicochemical parameters of the hot springs analyzed in this study.

**Supplementary Table S2** Pairwise average amino acid identities (AAI) of the 11 hot spring comammox *Nitrospira* MAGs and three reference comammox *Nitrospira* MAGs (*N. inopinata*, *Ca*. N. nitrificans, and *Ca*. N. nitrosa). AAI and minimum aligned protein number are indicated by orange and blue boxes, respectively.

**Supplementary Fig. S1** Phylogenetic analysis of AmoCAB and NxrAB protein families. (**a**) AmoCAB-based maximum likelihood tree showing the affiliation of the 11 hot spring comammox MAGs with 38 reference genomes of clade A (highlighted by yellow background) and clade B (green background) *Nitrospira*. Thaumarchaeota archaeon SCGC AAA799-D11 was used as an outgroup. Bootstrap support (based on 1000 iterations) ≥ 70 and ≥ 90 is indicated by gray and black circles, respectively. (**b**) NxrAB-based maximum likelihood tree showing the affiliation of the 11 hot spring comammox MAGs with one reference genome of sublineage I *Nitrospira* (blue background) and four reference genomes of sublineage II *Nitrospira* (yellow background). Bootstrap values (based on 1000 iterations) ≥ 70 and ≥ 90 are indicated by gray and black circles, respectively. The identifiers of comammox *Nitrospira* MAGs obtained from hot springs are shown in red.

**Supplementary Fig. S2** Schematic representation showing the arrangement of urease operons in selected comammox *Nitrospira*. The predicted functions of the encoded proteins are represented by colors and protein tags.

**Supplementary Fig. S3** Schematic overview of subtypes of energy-converting hydrogenase-related complexes (Ehr) identified in selected comammox *Nitrospira*. Homologous encoded proteins are indicated by colors and protein tags.

**Supplementary Fig. S4** Transcriptional rank of functional genes of two representative hot spring comammox *Nitrospira* MAGs: (**a**) gene expression of SRBZ-2_Bin320 in the sample QQ; and (**b**) gene expression of JZ-4_Bin17 in the sample JZ-2. Displayed are the 250 most active genes from the community-wide metatranscriptomes. Coloured bars and corresponding labels highlight the key genes related to nitrogen metabolism, thermal adaptation and oxidative stress.

**Supplementary Fig. S5** Analysis of amino acid usage by 12 thermal and 18 non-thermal clade A comammox *Nitrospira*. (**a**) Heat map of the amino acid usage by thermal (11 from hot springs in the present study and 1 from a hot water pipe) and non-thermal clade A comammox *Nitrospira* MAGs. The color code indicates the relative abundance of each amino acid after z-score normalization. (**b**) Comparison of specific amino acid usage proportions between genomes/MAGs in thermal and non-thermal groups. Statistical significance (*p* < 0.01) is indicated by asterisks.

**Supplementary Fig. S6** Comparisons of general genomic features between 12 thermal and 18 non-thermal Clade A comammox *Nitrospira*. (**a**) IVYWREL index; (**b**) predicted number of transmembrane transporters; (**c**) expected number of amino acids in transmembrane helices (ExpAA, only counted when ≥ 18 residues are predicted to be within the membrane-spanning region); (**d**) percentage of functional gene annotations (calculated by dividing the number of annotated coding sequences by the number of all scaffolds, multiplied by 100).
**Supplementary Fig. S7** The genome size of 12 thermal and 18 non-thermal Clade A comammox *Nitrospira* plotted against their OGT (Pearson’s correlation *p*= 1.68e-07).

**Supplementary Fig. S8** Presence or absence of specific metabolic or biosynthetic capacities in comammox *Nitrospira* genomes/MAGs obtained from thermal and non-thermal habitats. The white box indicates pathway/gene absence, while the colored box indicates pathway/gene presence. The major metabolic or biosynthetic capacities (rows) are shown for 12 thermal and 18 non-thermal clade A comammox *Nitrospira* MAGs (columns). Details of the metabolic capacities are provided in Supplementary Dataset 2.

***Text S1 Comparative genomics*.** As the genome completeness will affect the parameters such as genome size and gene count, we set a threshold of 85% completeness for the selection of comammox *Nitrospira* MAGs for comparison in order to minimize the potential bias. After removing the low-quality genomes with completeness below 85% and contamination above 5%, a total of 30 comammox *Nitrospira* MAGs belonging to comammox clade A were used for comparative genomic analysis, including 11 from the present study and 19 downloaded from the NCBI-Refseq database (Supplementary Dataset 4). To unveil genomic differences, comammox MAGs were manually classified into two groups according to the temperature of the habitat where they were obtained. Specifically, 11 hot spring comammox *Nitrospira* from the present study and one assembled from a hot water pipe (*N. inopinata*) were classified as thermal comammox *Nitrospira*, while the remaining 18 MAGs were derived from non-thermal habitats. Genome qualities of these 30 comammox MAGs were evaluated using CheckM. General genomic features including genome size, gene count, average gene length, coding density (i.e., the total length of genes divided by the total length of genome multiplied by 100) and GC content between the thermal and non-thermal comammox MAGs were statistically analyzed by conducting the Wilcoxon rank sum test implemented in the “WilcoxCV” package and visualized by generating box plots using the “ggplot2” package in R v4.0.5 ^1^. The optimal growth temperature (OGT) was calculated based on the command line tool Tome (Temperature optima for microorganisms and enzymes) with details described in Li et al. 2019 ^2^.

***Text S2 Habitat distribution of thermophilic comammox Nitrospira.*** 16S rRNA gene sequences were identified using RNAmmer v1.2 ^3^. To examine the widespread of target genomes among thermal habitats, the representative 16S rRNA gene sequence, *amoA* and *nxrA* gene sequences retrieved from SRBZ-2_Bin320 were used to search against NCBI-nr/NCBI-nt databases. Only hits derived from thermal habitats with sequence identities ≥ 97% for 16S rRNA and ≥ 95% for functional genes were kept.

**Supplementary Table S1** Physicochemical parameters of the hot springs analyzed in this study.

| Sample | MAG/Metatranscriptomic | Hot spring | Date | Latitude | Longitude | pH | T (℃) | DO (mg/L) | Salinity (g/kg) | EC (ms/cm) | NH_4_^+^-N (mg/kg) | NO_2_^-^-N (mg/kg) | NO_3_^-^-N (mg/kg) | SO_4_^2-^-S (mg/kg) | Organic carbon (g/kg) | PO_4_^3-^ (mg/kg) |
| --- | --- | --- | --- | --- | --- | --- | --- | --- | --- | --- | --- | --- | --- | --- | --- | --- |
| DGJ01_4_201608 | DGJ01_4_Bin137 | DGJ | 2016.08 | 29^0^60'14'' | 85^0^74'84'' | 8.24 | 80 | 0.09 | NA | 1.91 | 0.04 | NA | NA | NA | NA | NA |
| DGJ02_3_201608 | DGJ02_3_Bin65 | DGJ | 2016.08 | 29°60'14'' | 85°74'84'' | 3.00 | 37 | 1.10 | NA | 0.44 | 6.10 | NA | NA | NA | NA | NA |
| QZM_B4_3_201608 | QZM_B4_3_Bin89 | QZM | 2016.08 | 28°24'85'' | 91°80'34'' | 7.00 | 60 | NA | NA | 2.31 | 0.53 | NA | NA | NA | NA | NA |
| QZM_B4_2_201608 | QZM_B4_2_Bin429 | QZM | 2016.08 | 28°24'85'' | 91°80'34'' | 7.00 | 60 | NA | NA | 2.31 | 0.53 | NA | NA | NA | NA | NA |
| JZ-3_201803 | JZ-3_Bin7 | JZ | 2018.03 | 25°26'28'' | 98°27'36'' | 8.00 | 64 | NA | NA | NA | NA | NA | NA | NA | NA | NA |
| JZ-4_201709 | JZ-4_Bin17 | JZ | 2017.09 | 25°26'28'' | 98°27'36'' | 6.50 | 38 | NA | 1.40 | 0.16 | 4.66 | 1.55 | 1.53 | 83 | 0.00 | 4.36 |
| JZ-4_201803 | JZ-4_Bin198 | JZ | 2018.03 | 25°26'28'' | 98°27'36'' | 8.00 | 52 | NA | 2.15 | 0.51 | 3.88 | 0.49 | 2.91 | 187 | 12.29 | 6.70 |
| JZ-4_201808 | JZ-4_Bin299 | JZ | 2018.08 | 25°26'28'' | 98°27'36'' | 8.00 | 52 | NA | 1.40 | 0.16 | 4.66 | 1.35 | 9.65 | 83 | 3.21 | 4.36 |
| SRBZ-2_201901 | SRBZ-2_Bin184, SRBZ-2_Bin320 | SRBZ | 2019.01 | 24°57'0'' | 98°26'14'' | 7.04 | 45 | NA | 2.01 | 0.45 | 0.78 | 8.36 | 1.65 | 379 | 2.04 | 0.20 |
| SZTDM-2_201709 | SZTDM-2_Bin289 | SZTDM | 2017.09 | 24°57'0'' | 98°26'0'' | 7.00 | 54 | NA | 2.46 | 0.25 | 16.31 | 1.54 | 1.55 | 66 | 0.00 | 5.93 |
| JZ-2_202101 | Metatranscriptomic | JZ | 2021.01 | 25°26'28'' | 98°27'36'' | 7.47 | 36 | NA | 2.44 | 0.12 | 12.10 | 0.81 | 6.05 | 223 | 5.04 | 0.52 |
| JZ-4_202101 | Metatranscriptomic | JZ | 2021.01 | 25°26'28'' | 98°27'36'' | 8.64 | 27 | NA | NA | 1.81 | NA | NA | NA | NA | NA | NA |
| QQ_202207 | Metatranscriptomic | QQ | 2022.07 | 24°57'1'' | 98°26'11'' | 6.80 | 70 | NA | NA | NA | 7.26 | NA | 4.84 | 104 | NA | 6.51 |

NA: Not available. DO was measured from the pore water of sediments.

**Supplementary Table S2** Pairwise average amino acid identities (AAI) of the 11 hot spring comammox *Nitrospira* MAGs and three reference comammox *Nitrospira* MAGs (*N. inopinata*, *Ca*. N. nitrificans, and *Ca*. N. nitrosa). AAI and minimum aligned protein number are indicated by orange and blue boxes, respectively.


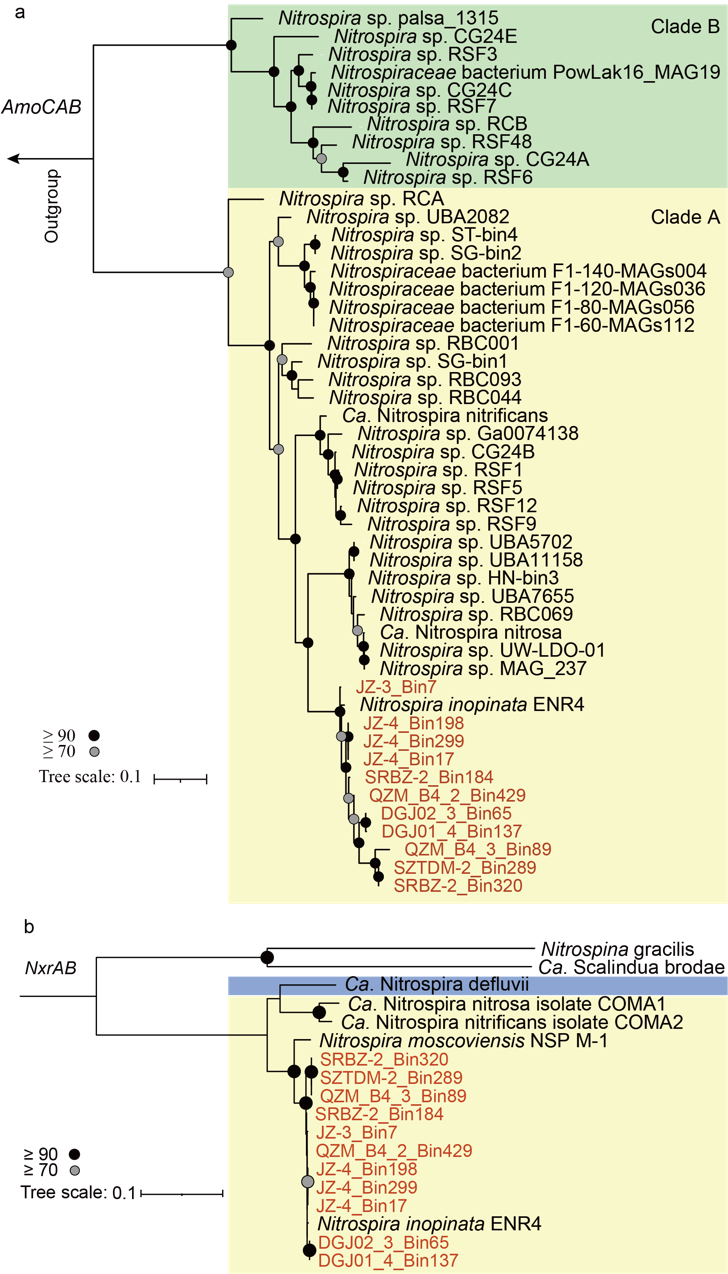


**Supplementary Fig. S1** Phylogenetic analysis of AmoCAB and NxrAB protein families. (**a**) AmoCAB-based maximum likelihood tree showing the affiliation of the 11 hot spring comammox MAGs with 38 reference genomes of clade A (highlighted by yellow background) and clade B (green background) *Nitrospira*. Thaumarchaeota archaeon SCGC AAA799-D11 was used as an outgroup. Bootstrap support (based on 1000 iterations) ≥ 70 and ≥ 90 is indicated by gray and black circles, respectively. (**b**) NxrAB-based maximum likelihood tree showing the affiliation of the 11 hot spring comammox MAGs with one reference genome of sublineage I *Nitrospira* (blue background) and four reference genomes of sublineage II *Nitrospira* (yellow background). Bootstrap values (based on 1000 iterations) ≥ 70 and ≥ 90 are indicated by gray and black circles, respectively. The identifiers of comammox *Nitrospira* MAGs obtained from hot springs are shown in red.


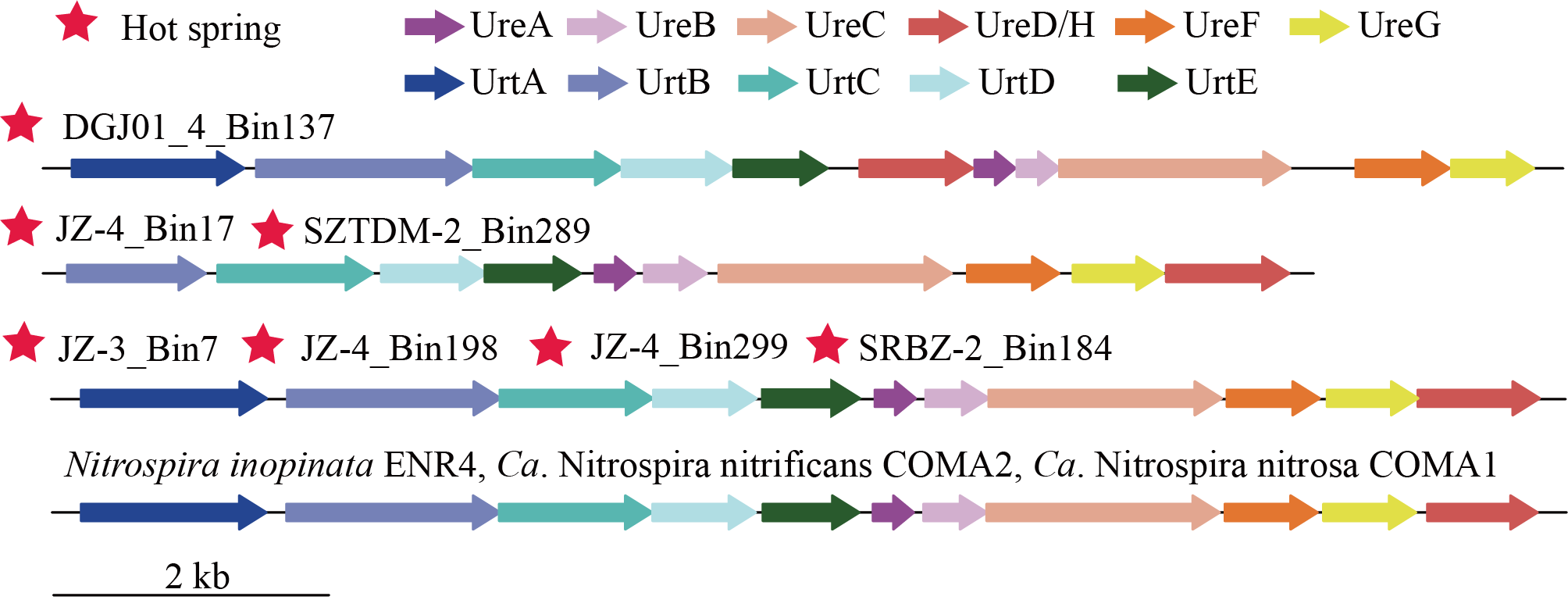


**Supplementary Fig. S2** Schematic representation showing the arrangement of urease operons in selected comammox *Nitrospira*. The predicted functions of the encoded proteins are represented by colors and protein tags.


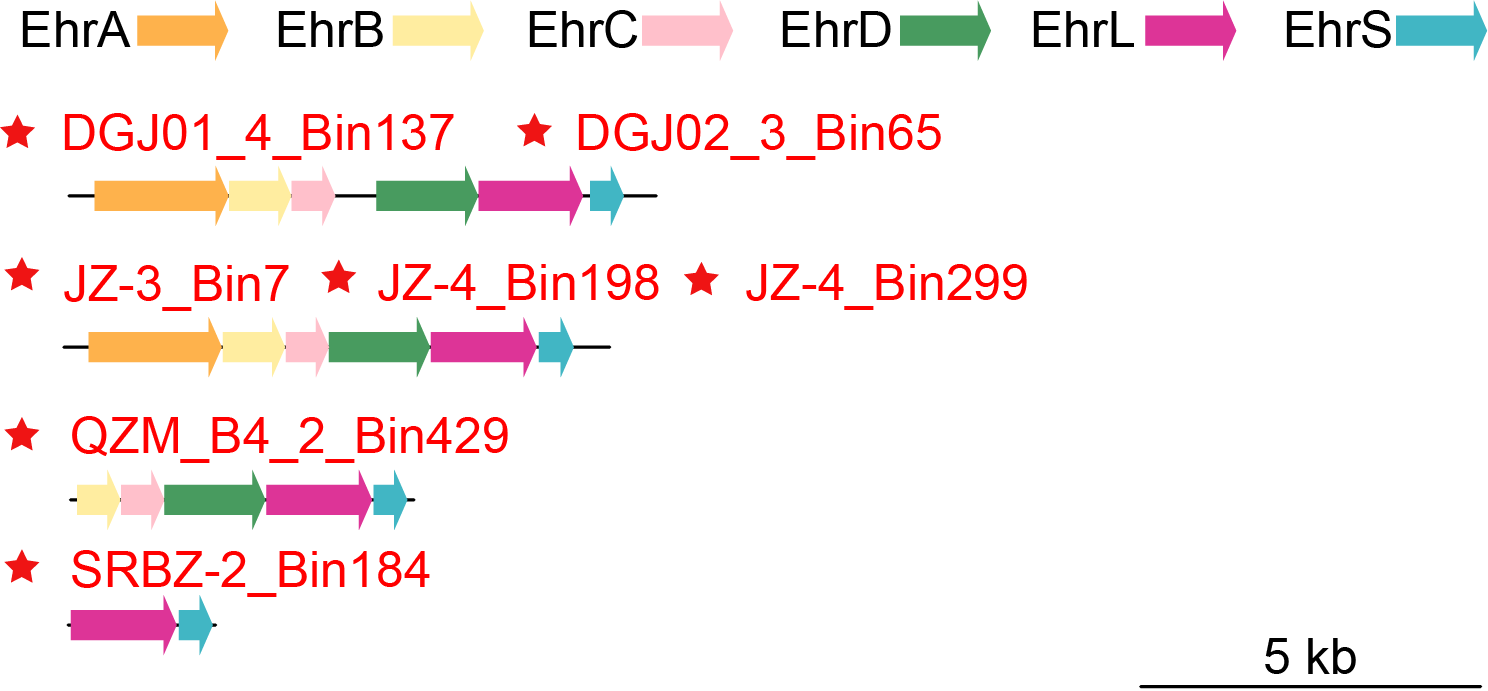


**Supplementary Fig. S3** Schematic overview of subtypes of energy-converting hydrogenase-related complexes (Ehr) identified in selected comammox *Nitrospira*. Homologous encoded proteins are indicated by colors and protein tags.


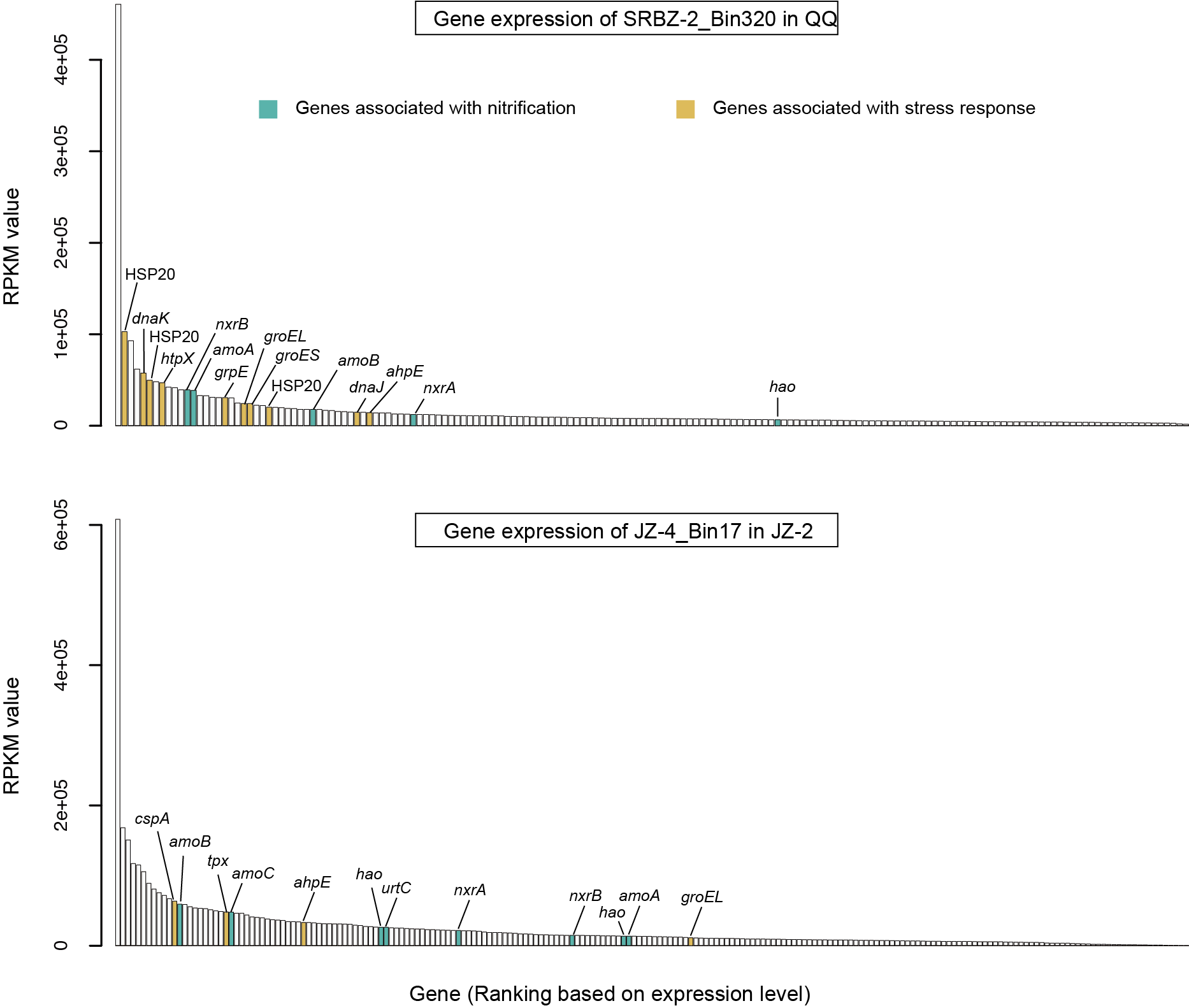


**Supplementary Fig. S4** Transcriptional rank of functional genes of two representative hot spring comammox *Nitrospira* MAGs: (**a**) gene expression of SRBZ-2_Bin320 in the sample QQ; and (**b**) gene expression of JZ-4_Bin17 in the sample JZ-2. Displayed are the 250 most active genes from the community-wide metatranscriptomes. Colored bars and corresponding labels highlight the key genes related to nitrogen metabolism, thermal adaptation and oxidative stress.


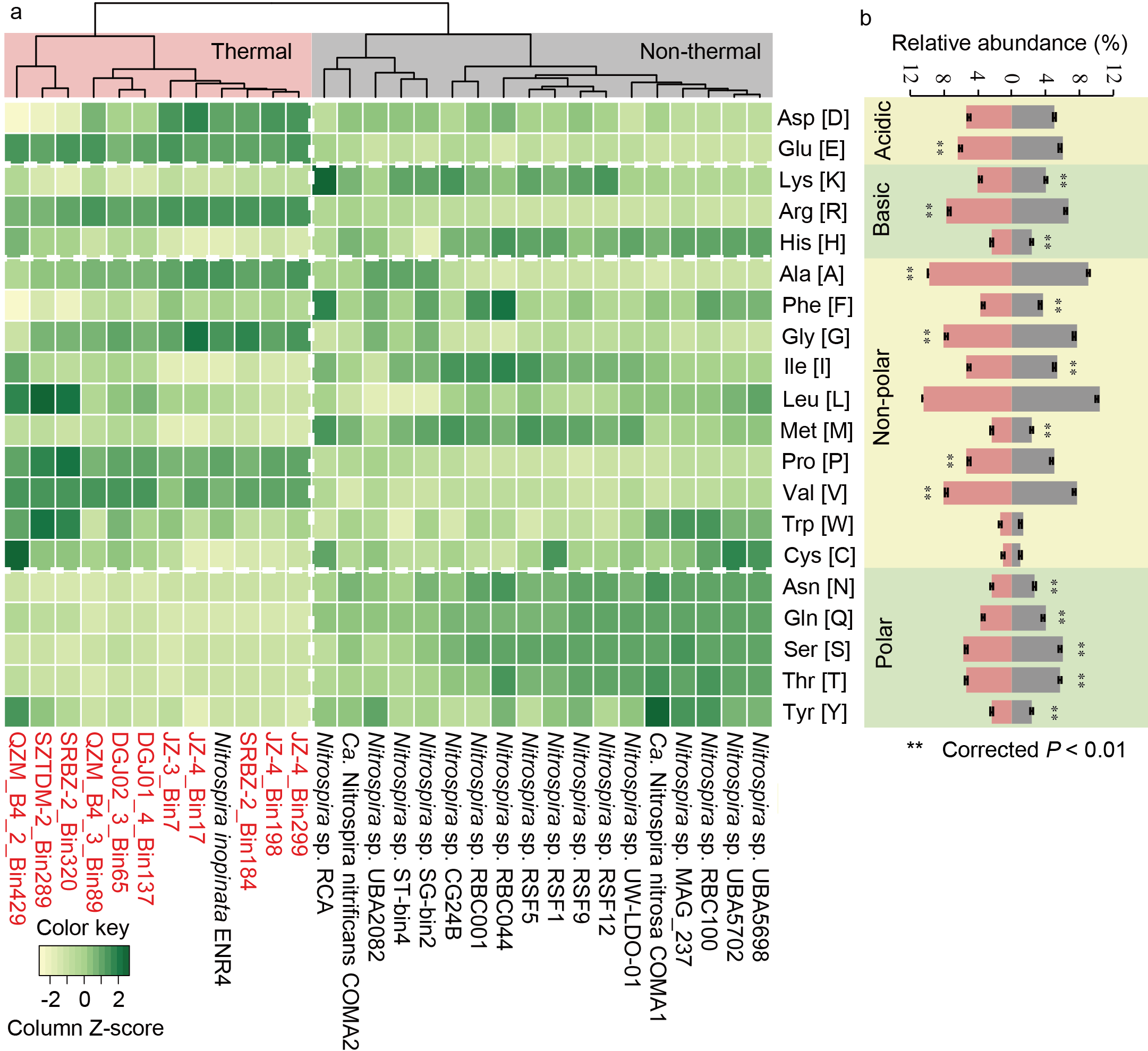


**Supplementary Fig. S5** Analysis of amino acid usage by 12 thermal and 18 non-thermal clade A comammox *Nitrospira*. (**a**) Heat map of the amino acid usage by thermal (11 from hot springs in the present study and 1 from a hot water pipe) and non-thermal clade A comammox *Nitrospira* MAGs. The color code indicates the relative abundance of each amino acid after z-score normalization. (**b**) Comparison of specific amino acid usage proportions between genomes/MAGs in thermal and non-thermal groups. Statistical significance (*p* < 0.01) is indicated by asterisks.


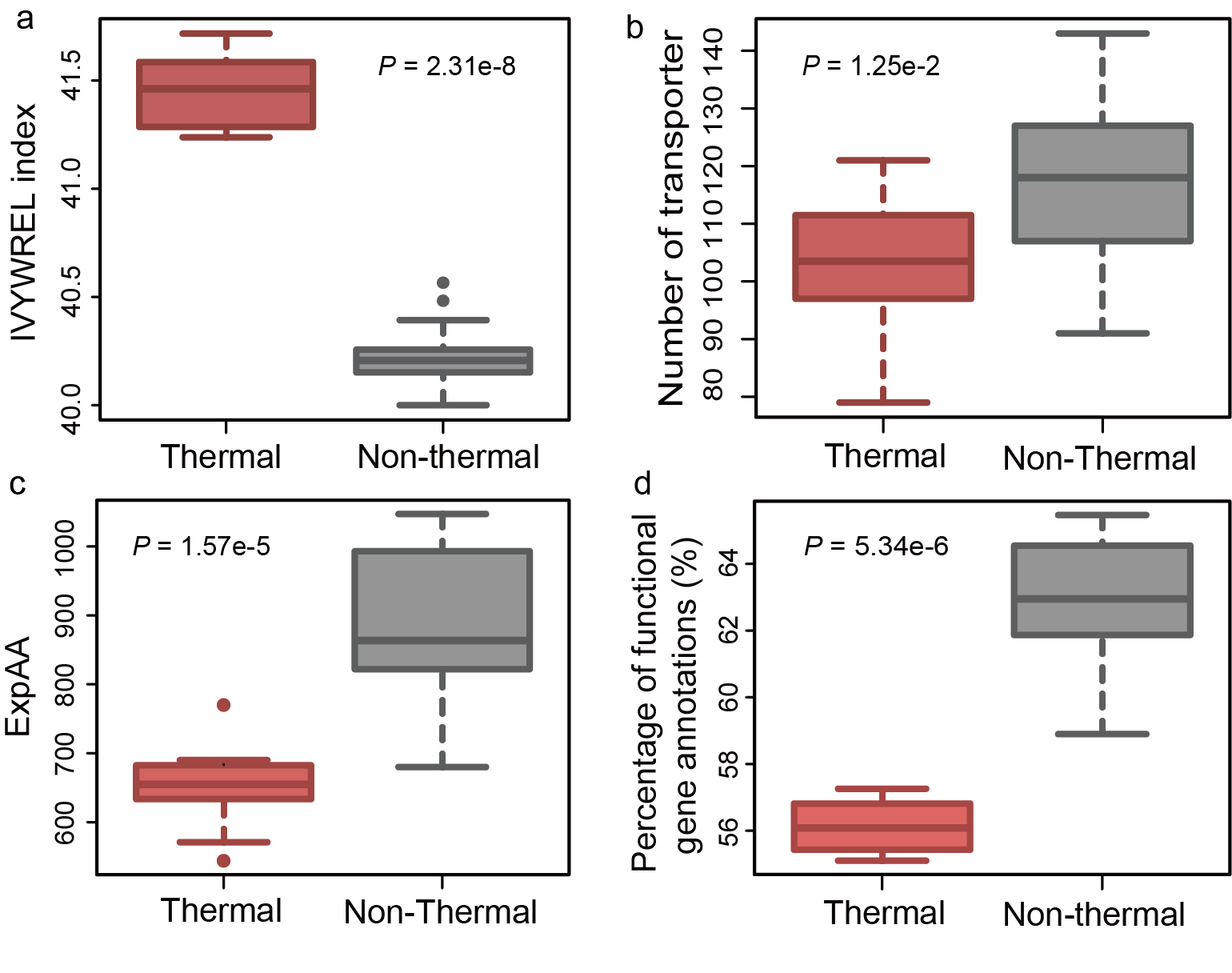


**Supplementary Fig. S6** Comparisons of general genomic features between 12 thermal and 18 non-thermal Clade A comammox *Nitrospira*. (**a**) IVYWREL index; (**b**) predicted number of transmembrane transporters; (**c**) expected number of amino acids in transmembrane helices (ExpAA, only counted when ≥ 18 residues are predicted to be within the membrane-spanning region); (**d**) percentage of functional gene annotations (calculated by dividing the number of annotated coding sequences by the number of all scaffolds, multiplied by 100).


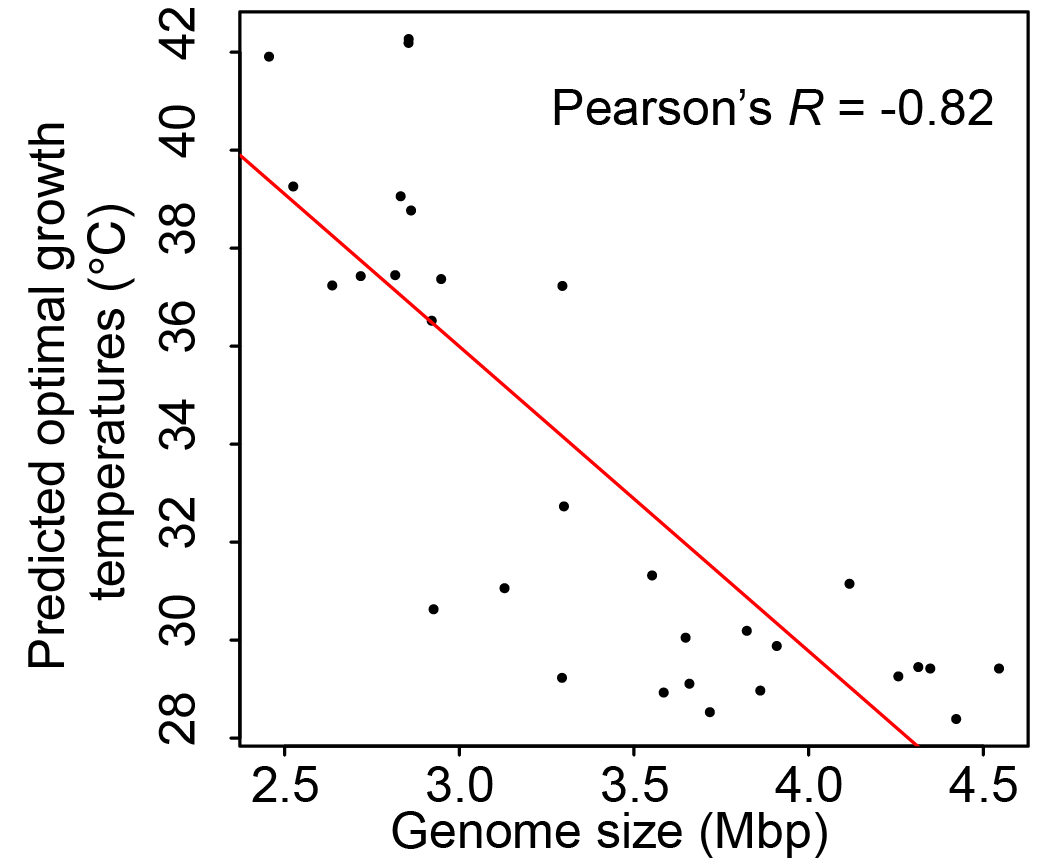

**Supplementary Fig. S7** The genome size of 12 thermal and 18 non-thermal Clade A comammox *Nitrospira* plotted against their OGT (Pearson’s correlation *p* = 1.68e-07).

**Supplementary Fig. S8** Presence or absence of specific metabolic or biosynthetic capacities in comammox *Nitrospira* genomes/MAGs obtained from thermal and non-thermal habitats. The white box indicates pathway/gene absence, while the colored box indicates pathway/gene presence. The major metabolic or biosynthetic capacities (rows) are shown for 12 thermal and 18 non-thermal clade A comammox *Nitrospira* MAGs (columns). Details of the metabolic capacities are provided in Supplementary Dataset 2.

1 Wickham H. in *ggplot2* 189-201 (Springer, 2016).

2 Li G, Rabe KS, Nielsen J, Engqvist MK. Machine learning applied to predicting microorganism growth temperatures and enzyme catalytic optima. *ACS synthetic biology.* 2019; 8: 1411-1420.

3 Lagesen K*, et al.* RNAmmer: consistent and rapid annotation of ribosomal RNA genes. *Nucleic Acids Res.* 2007; 35: 3100-3108.
